# *Fusarium* Molds and Mycotoxins: Potential Species-Specific Effects

**DOI:** 10.3390/toxins10060244

**Published:** 2018-06-15

**Authors:** Alessia Bertero, Antonio Moretti, Leon J. Spicer, Francesca Caloni

**Affiliations:** 1 Department of Veterinary Medicine (DIMEVET), Università degli Studi di Milano, Via Celoria 10, 20133 Milan, Italy; alessia.bertero@unimi.it (A.B.); francesca.caloni@unimi.it (F.C.); 2 Institute of Sciences of Food Production, National Research Council of Italy, Via Amendola 122/O, 70126 Bari, Italy; 3 Department of Animal and Food Sciences, Oklahoma State University, Stillwater, OK 74078, USA; leon.spicer@okstate.edu

**Keywords:** *Fusarium* mycotoxins, species-specificity, beauvericin, deoxynivalenol, enniatin B fumonisin B_1_, molds, zearalenone

## Abstract

This review summarizes the information on biochemical and biological activity of the main *Fusarium* mycotoxins, focusing on toxicological aspects in terms of species-specific effects. Both in vitro and in vivo studies have centered on the peculiarity of the responses to mycotoxins, demonstrating that toxicokinetics, bioavailability and the mechanisms of action of these substances vary depending on the species involved, but additional studies are needed to better understand the specific responses. The aim of this review is to summarize the toxicological responses of the main species affected by *Fusarium* mycotoxins.

## 1. Introduction

Mycotoxins are secondary metabolites produced, under opportune environmental conditions, by filamentous fungi, and particularly by microfungi of the genus *Fusarium*. These substances, characterized by a low molecular weight, can cause a wide range of diseases as well death in humans and other animals. *Fusarium* mycotoxins are world-spread contaminants naturally occurring in commodities, food and feed [1,2,3,4,5,6]. The majority of mycotoxicoses result from eating contaminated foods, but skin contact and inhalation of toxins are also sources of exposure [7]. The occurrence of mycotoxins, alone and in combination, represents a real risk for human and animal health. In this context, further studies are needed to investigate the potential effects of these compounds, alone and in combination, and their species-specific effects, since it has been demonstrated by several authors that toxicokinetics, bioavailability and the mechanisms of action of these substances vary depending on the species involved [8,9,10].

Moreover, even though the animal and human exposure is well established [11,12,13,14,15,16], data regarding the incidence of mycotoxicoses are difficult to obtain and no limits have been identified by the authorities for some of these compounds up to now (e.g., enniatins, beauvericin). The European Commission questioned the European Food Safety Authority (EFSA) for a scientific opinion concerning the risks for human and animal health related to the presence of beauvericin and enniatins in food and feed, but, because of the lack of relevant toxicity data, they were unable to do a proper risk assessment. However, they concluded that, even if the primary source of these mycotoxins for animals are cereal grains and derived products, which are the principal ingredients of many animal diets, acute mycotoxicoses due to an exposure to beauvericin and enniatins is unlikely, both for farm and companion animals.

Data regarding chronic exposure in chickens showed the same results, but, to extend this consideration to other animal species, due to the species-specificity of these compounds, further studies for the evaluation of LOAELs/NOAELs are necessary in order to be able to estimate the potential risk for a chronic exposure to these emerging mycotoxins.

This review focuses on the main *Fusarium* mycotoxins analyzing the potential species-specific effects of these compounds.

## 2. Fumonisin B_1_

Fumonisins are a group of mycotoxins mainly produced by *Fusarium verticillioides* and *Fusarium proliferatum* that occur worldwide primarily in maize, especially when cultivated in warm regions [17,18], where they cause the so-called *Fusarium* Ear Rot (FER). Moreover, several other *Fusarium* species of lower importance for their spread, incidence, and worldwide occurrence on agro-food crops are able to produce this mycotoxin [19].

A lot of fumonisin homologues are known (at least 28) [20], but the most important group of fumonisins is the B series, which include fumonisin B_1_ (FB1), B_2_ (FB2), B_3_ (FB3) [21]. In 2002, the International Agency for Research on Cancer (IARC) classified FB1 as possibly carcinogenic to humans (group 2B) [22,23,24] and several studies reported that FB1 is associated with an increased prevalence of esophageal [24,25,26,27,28] and liver cancer in humans [29]. Moreover, this mycotoxin has been found to exert a toxic action against several organs and apparatuses (nervous and cardiovascular systems, liver, lung, kidney) in animals [18,25,30,31,32,33,34]. The responses induced by FB1 are species- and gender-specific: for example, FB1 can induce hepatocarcinoma [35] and nephrocarcinoma in rats [34,36], but were not related to esophageal cancer in animals [37].

The complex mechanism of action of FB1 is based on the inhibition of sphingosine (sphinganine) N-acetyltransferase (ceramide synthase). The fumonisins that are characterized by an unsubstituted primary amino group at C2 are structurally close to sphinganine and sphingosine and cause the inhibition of the ceramide synthase. This leads to the disruption of the ceramide biosynthesis with the accumulation of intermediates (sphingoid bases) of sphingolipid biosynthesis [18,38,39,40,41] and the consequential alteration of the sphingolipid metabolism in tissues [18,38,42,43,44,45]. FB1 is a competitive inhibitor with respect to both substrates of ceramide synthase (sphinganine, coming from the *de novo* synthesis, and sphingosine from sphingolipid turnover) [18,43,44]. This inhibition leads to the blockage of complex sphingolipid biosynthesis, essential for cell regulation and to the accumulation of sphinganine and, to a lesser degree, sphingosine [42,43,44,45], in tissues, blood, and urine in vivo but also in vitro. Moreover, the ratio of free sphingoid bases (sphinganine/sphingosine–Sa/So) increases in blood, several tissues (e.g., lung, liver, intestine) and in cultured cells [46,47,48]. Therefore, inhibition of ceramide synthase by FB1 causes a rapid increase of the intracellular concentration of sphinganine and, to a lesser extent, sphingosine and their 1-phosphate metabolites. Generally, sphingosine accumulation takes place when cell damage causes the disruption of the membrane integrity and membrane degradation starts [49].

Sphingolipids strongly influence cell behavior because they play fundamental roles for the maintenance of membrane integrity and structure and act as precursors for second messengers that mediate the cellular response to growth factors [50]. Moreover, many works demonstrated an inhibitory action on cell growth and a cytotoxic effect for ceramide and sphingosine, while sphingosine phosphate has mitogenic and anti-apoptotic effects [51]. Thus, the perfect regulation of sphingolipid turnover and biosynthesis pathways is very important and the perturbing action of fumonisins can deeply affect the organism.

Fumonisins have been found to commonly occur in cereal grains and animal feed in combination with other *Fusarium* mycotoxins including beauvericin (BEA), a so-called emerging mycotoxin [16,52]. Therefore, interactions between fumonisins and other mycotoxins should be the focus of future studies.

### 2.1. Species-Specific Effects

#### 2.1.1. Horses

Horses are the most sensitive species to FB1 toxicity, and FB1 concentrations of 0.02 and 0.12 μg/g in feed are already able to cause outbreaks in this species [53], even if the concentration range found in outbreaks is wide, from 0.1 to 126 μg/g [54]. The neurotoxic (leukoencephalomalacia—Equine Leuko Encephalo Malacia (ELEM)) and the hepatotoxic forms are the two syndromes, which can occur simultaneously or independently, described in horses with FB1-mycotoxicoses. Central nervous system, liver and heart are involved as target organs. ELEM typical signs are: sweating, inability to swallow, muscle fasciculation and weakness, in coordination and ataxia, hypermetria, circling, head pressing, tonic-clonic seizures, paresis, hyperexcitability or depression, blindness, dilated pupils, and absence of a pupillary light reflex [53,55]. Early moderate neurological signs such as tongue paralysis and ataxia may precede by hours or days the onset of more severe signs. The ELEM is characterized by a rapid clinical course (hours or days) and a high mortality. Sometimes, death can occur without sign of disease. The histopathological findings are mainly a liquefactive necrosis with an influx of macrophages of the white matter of the cerebral hemispheres with edema and hemorrhage [18]. Equines are the only species that suffer from this neurological syndrome related to FB1 intoxication. The hepatotoxic syndrome, which is less frequent than the neurotoxic form, is characterized by hyporexia, depression, icterus and edema of the head. The main histopathological sign is a centrilobular necrosis with periportal fibrosis [18].

Regarding the most recently recognized cardiotoxicity, Smith and colleagues documented for the first time in 2002 that horses with fumonisin B_1_-induced neurologic syndrome have cardiovascular dysfunction. In a study performed by these authors, in which FB1 was administered intravenously (0.20 mg/kg), signs included decreased cardiac output, negative chronotropic and inotropic effects and a reduction in the arterial pulse pressure. Moreover, all the animals showed an increased sphingosine and sphinganine concentration in the myocardium [56]. Although the mechanism of action of fumonisins is not completely understood, it has been demonstrated that high concentrations of sphingosine inhibit L-type calcium channels in several mammalian species myocardial cells [57,58,59,60], leading to a decrease in Ca ion release with a reduction of cardiac activity. It can also be hypothesized that the development of leukoencephalomalacia may be related to the decreased cardiovascular function with cerebral vessel damage.

A study was performed in vitro on primary isolated epidermal and dermal hoof cells to evaluate the influence of FB1 on the lamellar tissue and sphingolipid metabolism [61]. A significant increase in sphinganine concentrations was found in the supernatant of the explants with a simultaneous decrease in the lamellar integrity, confirming in vitro the in vivo findings.

Regarding the reproductive toxicity, effects were observed only at doses that induce clear clinical signs in the mares [62]. In vitro toxicity of fumonisin B_1_ on fresh and frozen-thawed semen has also been assessed. Sperm viability and motility, chromatin stability and reactive oxygen species (ROS) production was assessed in a study by Minervini et al. [63]. No effects on viability were found in fresh sperm after exposure to FB1 up to 25 µM while reduction of total and progressive motility was recorded at a concentration similar to those reported as neurotoxic (10 µg/g). Just one frozen-thawed sample showed chromatin damage after FB1 exposure. Moreover, the study found that the action of FB1 on sperm functional parameters was subject-dependent.

#### 2.1.2. Ruminants

Ruminants appear to be considerably less sensitive to FB1 than horses and pigs and, in cases of highly contaminated feed, some animals may develop mild biochemical alterations and microscopic liver (diffuse mild hydropic degeneration in a periacinar pattern) [64] and renal lesions [65]. In a study by Osweiler and colleagues [64], serum liver enzymes showed a mild elevation after consumption for 10 days of a contaminated feed (148 microg/g): changes were too small to be suggestive of severe liver disease, but they suggested an effect of the mycotoxin on hepatocyte and biliary excretion. Moreover, after a chronic feeding period, lymphocyte blastogenesis was impaired.

Studies suggested that the low sensitivity of ruminants to FB1 may be due to its low bioavailability after oral administration [66] and, contrarily to other mycotoxins, FB1 does not undergo rumen microbial degradation and passes the rumen virtually unchanged [67]. Moreover, FB1 does not affect the production of short-chain fatty acids in rumen, so this mycotoxin had no toxic effects on the ruminal microflora [68].

Reproductive effects of FB1, alone and in combination with other mycotoxins, have been investigated using bovine granulosa cell models. When tested alone, FB1 did not show effects on granulosa cell proliferation and had no significant effect on progesterone production at any dose, whereas at a concentration between 1 and 3 µM weakly inhibited estradiol production [69], thus demonstrating that FB1 may impair reproductive function in cattle. The decrease in estradiol production was not linked to a significant modification on CYP19A1 expression in bovine granulosa cells, while it was observed in porcine granulosa cells [70], indicating that another mechanism of action should be implied (i.e., estradiol metabolism and/or FSH/IGF1-receptors). It has been reported that FB1 can modulate the endocrine system by antagonism of nuclear receptor transcriptional activity [71], but whether this is how FB1 is acting in granulosa cells will require further elucidation. Moreover, many in vitro studies concerning the reproductive effects in cattle have shown that the co-exposure plays a fundamental role, and that doses tested have a strong influence on the entity and type of exerted effects [72,73].

#### 2.1.3. Pigs

Pigs are, together with horses, the most sensitive species to fumonisins. A typical and species-specific presentation of FB1 acute mycotoxicosis in pigs is pulmonary edema, a sign that has been reported only in this species. It was described for the first time in 1981 after the experimental administration of feed contaminated by *F. verticilloides* [65]. After that, in 1989, an outbreak due to consumption of corn contaminated by fumonisins resulted in the deaths of several pigs. The main sign was a huge pulmonary edema, thus the syndrome was called “porcine pulmonary edema” (PPE), whereas some animals also showed reproductive anomalies (e.g., abortion).

Further studies were able to induce the same pulmonary edema by administering orally and intravenously purified FB1 [74,75]. The pathogenesis of the toxicosis-induced pulmonary edema is linked primarily to an increase in pulmonary capillary hydrostatic pressure due to an acute left-sided heart failure and to an increase in the vascular permeability caused by damages to the alveolar capillary endothelium and alveolar epithelium. Other signs of fumonisin toxicosis are hepatic, cardiovascular and immune system damages together with the alteration of the sphingolipid metabolism. Development of lethal pulmonary edema is reported in pigs fed with concentrations of 16 mg/kg of FB1 for 4–7 days or intravenously injected [31]. Clinical signs are: inactivity, respiratory distress shown by increased respiratory rate, abdominal respiratory effort and open mouth breathing, and a decreased heart rate, which occur during the 12-h period that precedes pulmonary edema and death [76]. In some cases, gastrointestinal signs have also been reported.

The cardiovascular signs include a negative chronotropic and inotropic effect, and the relaxation of the vascular smooth muscle that causes a reduction of the cardiovascular reserve, effects that are probably mediated via the increase in sphingosine concentration that inhibits the L-type calcium channels. The hepatic damage (characterized by cholestasis, hepatic necrosis, cell proliferation) is dose- and time-dependent and is characterized by an increase in the activity of the hepatic enzymes in serum, icterus, anorexia and hepatic encephalopathy.

In swine, as in the other species, the FB1 causes an inhibition of the ceramide synthase with a resultant increase in the sphingoid bases, sphinganine and sphingosine, in serum and tissues within 24 h [77], which is the earliest and most specific time- and dose-dependent sign of FB1 toxicosis [76]. Hypercholesterolemia also is a sensitive and specific parameter to detect FB1 exposure. Necropsy of pigs that died of PPE showed a pulmonary interstitial edema with widened interlobular septa in the lungs and the presence of modified transudate in the airways and in the thoracic cavity [77]. The major histopathological signs were presence of fluid in the connective tissue of bronchi, vessels, subpleural space and interlobular septa. No signs of inflammation were present. Some endothelial cells of lung vascular system appeared damaged and apoptotic and a dense accumulation of membranous material was found within the lung intravascular macrophages, which was probably the result of the phagocytosis of dead endothelial cells. This lesion is species-specific: comparable endothelial lesions were observed only in pigs [78]. 

In vitro studies were performed on porcine lung endothelial cell cultures to clarify the action of FB1. An association between the increase in albumin permeability detected within 12 h of treatment with FB1 and cell death was recorded, therefore the authors hypothesized that cell death played a role in the permeability increase [79]. Nevertheless, the in vitro increase in permeability was not considered physiologically relevant in vivo [79] because studies in pigs with PPE showed that only a few cells were affected by degeneration, and the number of involved cells was not sufficient to justify the huge increase in permeability. On the contrary, the significant time- and dose-dependent increase in sphingoid bases observed in lung and endothelial cells, in vivo and in vitro studies [77] was considered more relevant. It has been hypothesized that the described accumulation of membranous material in lung capillary is directly caused by the alteration of the sphingolipid metabolism induced by FB1 that acts on Golgi and endoplasmic reticulum causing an impairment of these cell organelles.

While PPE occurs only in case of high-level exposure, a chronic exposure to a lower concentration administered orally was found to be associated to a right ventricular hypertrophy and medial pulmonary arterial hypertrophy indicative of pulmonary hypertension [80]. Other lesions described with FB1 chronic intoxication include hyperplasia of the basal cell layer of the esophageal mucosa, sometimes with hyperplastic regions and gastric ulceration. These findings were particularly interesting because of the described association between FB1 ingestion and the occurrence esophageal cancer in humans [81].

Moreover, FB1 has been implicated in poor reproductive performance in pigs. In vitro studies were performed to analyze the effect of FB1 on the reproductive functions, using cultures of porcine granulosa cells. FB1 decreased the granulosa cell number with a dose 10–14 µM but had no effect at lower doses [70]. Comparable results were obtained with many porcine cell lines, such as renal (LLC-PK1) [82] and intestinal (IPEC-1) [83] cell lines and also in primary cells (i.e., porcine lymphocytes) [84]. Regarding the effects on granulosa cell steroidogenesis, FB1 stimulated progesterone production (not mediated by an increased activity of the mitochondrial enzyme cytochrome P450scc) while it had no significant effect on estrogen production [70].

## 3. Deoxynivalenol

Deoxynivalenol (DON) is a member of the mycotoxin family of trichothecene, which are potent inhibitors of proteic synthesis. This family constitutes the largest group of *Fusarium* mycotoxins, with more than 150 compounds. The basic structure is a tetracyclic ring system, and, based on the substituents, trichothecenes are grouped into different types (A–D). DON, also known as vomitoxin, is a common contaminant of wheat, and wheat-based products [85]. This mycotoxin is produced mainly by *F. graminearum*, and *F*. *culmorum*, and, at a lesser extent, by *F. cerealis* (syn. *F. crookwellense*), and *F. pseudograminearum*. All of these species commonly occur on wheat, but they can also occur on maize, where they can cause the accumulation of DON [86]. DON has been classified by the International Agency for Research on Cancer (IARC) in group 3 as it did not show carcinogenic actions in humans [87].

Alongside this major metabolite, there are also its acetylated and modified forms: the 3-acetyl-DON (3-Ac-DON), 15-acetyl-DON (15-Ac-DON) and DON-3-glucoside (DON3G). The latter is a modified form of DON in plants, also called masked DON, and represents the main plant metabolite of DON [88]. In particular, masked mycotoxins are mycotoxin derivatives generated via conjugation with other complex molecules such as sugars, amino acids, sulfate groups and many other biological components [89]. There is great concern over these matrix-associated or modified mycotoxins because they represent an analytical challenge and, moreover, these molecules can still retain toxic effects due to a reactivation that can occur in certain conditions, for example in the gastrointestinal environment [90].

The accumulation of DON and other trichothecenes in plants is the cause of a wheat disease, the *Fusarium* Head Blight, which causes severe loss of yield and reduces the quality of the wheat kernels [91]. Both acute and chronic toxicity due to ingestion of DON are reported. The acute syndrome is characterized by gastrointestinal signs such as abdominal discomfort, diarrhea, vomiting, and anorexia. The most frequent clinical signs of a chronic exposure to DON are weight loss and anorexia [92]. However, sensitivity and symptoms related to DON exposure vary according to the species involved.

The mechanism of action of this mycotoxin is based on the inhibition of protein synthesis in eukaryotes. The trichothecenes interact with peptidyl transferase enzyme binding the 60S ribosomal subunit, thus causing the inhibition of translation [93] and also ribotoxic stress. Another mechanism of action involves the activation of several mitogen-activated protein kinases (MAPKs), which are responsible for many effects of the DON (i.e., apoptosis, inflammatory response, oxidative stress, etc.) [94]. The main cellular targets of trichothecenes are the leucocytes: these mycotoxins can cause immunosuppression or immune stimulation [92]. Moreover, the potential of deoxynivalenol (DON) to act as endocrine disruptor has been investigated in previous studies and is an object of increasing interest [95,96] with focus on the action, in the different species, on follicular maturation and steroidogenesis.

### 3.1. Species-Specific Effects

#### 3.1.1. Pigs

Pigs, and particularly young animals, are poorly tolerant to DON [97]. The absorption of DON in pigs is generally high as well as distribution. DON and its metabolites were excreted primarily via urinary but also via biliary routes. In contrast to ruminants, only a minor metabolization of DON takes place in pigs and just a limited quantity of DON can be de-epoxidated by the gastrointestinal microflora [98]. The estimated dietary concentrations indicate that the risk of acute adverse effects from feed containing DON and its derived molecules is low, but a risk of chronic adverse effects is more relevant [88].

The most frequent signs described for pigs undergoing chronic intoxication (5–8 mg/kg) are a reduced feed intake and body weight gain [97]. Other clinical signs described in literature are lesions in stomach, intestine, lungs and kidneys. An alteration of the plasma biochemical parameters (plasma nutrients and enzyme activities) can also occur .In a study performed on twenty-four 5-week-old piglets subjected to chronic administration of DON, morphological and histological changes of the intestine were found (i.e., atrophy and fusion of villi, decreased cell proliferation in the jejunum) together with reduced jejunal and ileal goblet cell and lymphocyte numbers, and a decreased expression of junctional protein E-cadherin and occludin [99]. Moreover, altered cytokine (IL-1β, IL-6, IL-10, TNFα) production in the intestine and mesenteric lymph nodes has been found after DON exposure. These findings, together with other studies, indicate that DON-contaminated feed can alter the innate immune response in a piglet’s gut [100] and the whole immunologic system [101,102], but a relevant clinical impact has not been observed. At higher doses (reached in case of extremely contaminated feed with a concentration of DON of 20 mg/kg feed), DON is reported to induce vomiting, but acute intoxication with such high concentrations are not frequent.

Regarding the reproductive effects of DON, previous studies reported that a concentration of 10 µM affected the process of follicular maturation with a decrease of the reserve pool of follicles, resulting in a significant decrease in the number of normal follicles [103]. These results are in agreement with other studies, where porcine cumulus-oocyte complexes exposed to DON at 0.02, 0.2, or 2 μM showed an increase in cumulus cell death and degeneration, with a consequent significant reduction in the proportion of oocytes that reached metaphase II [104]. Concerning the effect of DON on granulosa cell proliferation and steroidogenesis in pigs, Ranzenigo et al. [105] reported increased granulosa cell numbers after treatment with 0.034 μM and 0.34 μM of DON and a drastic reductionin granulosa cell numbers with 3.4 μM of DON. Differently, Pizzo et al. [73] found that DON did not alter cell proliferation of bovine granulosa cells at concentrations ranging from 0.1 to 3.3 µM (see the next section).

Ranziego et al. [105] found that DON had biphasic effects on FSH plus IGF-I-induced estradiol production, increasing estradiol production by granulosa cells at smaller doses (10 ng/mL) and inhibiting at larger doses (100 and 1000 ng/mL) while DON had an inhibitory effect at 100 and 1000 ng/mL on progesterone production. Moreover, larger doses (1000 ng/mL) of DON inhibited CYP19A1 and CYP11A1 gene expression induced by FSH and IGF-I.Summarizing, DON showed direct dose-dependent effects on granulosa cell steroidogenesis and proliferation, with a direct ovarian effect that couldimpact reproductive performance in swine.

#### 3.1.2. Ruminants

Compared to pigs, the bovine species is considered less sensitive to DON due to the metabolism by rumen microbes. In this species, DON is almost totally transformed to the less toxic de-epoxidised metabolite DOM-1 by the ruminal flora and only traces of DON (<1%) are absorbed and found in the systemic circulation. However, it should be considered that, while in healthy ruminants DON is converted into DOM-1, in ruminants with acidosis or in young animals, in which the ruminal system is not fully efficient, the toxicokinetics of DON could be different and these animals could be more susceptible to the toxic effects of this mycotoxin. The renal route is the main route of excretion of this toxicant.

The CONTAM Panel identified a NOAEL for dairy cows and steers of 5 to 18 mg DON/kg feed, respectively, since no adverse effects on body weight, feed intake, milk yield, etc. were observed in a period of 13 weeks [88]. The feed concentrations derived from the sum of DON and its metabolites for ruminants were below the indicated NOAELs for chronic adverse effects, thus they are not so frequent. In North Europe, a toxic syndrome characterized by an increase in inflammatory reactions with mastitis and laminitis was related to exposure to grass silage highly contaminated by DON. However, it was not clear if the symptoms were caused only by DON or if other trichothecenes were involved [106].

In a study performed by Charmley et al. [107], dairy cows feed with 0.59, 42, and 104 mg DON did not show a change in feed intake and total milk output was not affected while milk fat decreased quadratically in relation to the DON concentrations. In another study [108], the effect of DON on the immune system (a target organ for DON in many species) was investigated. Fourteen Holstein cows were fed with a diet contaminated with DON for 18 weeks (4.6 mg DON/kg dry matter). At the end of the study period, the peripheral blood mononuclear cells were slightly less viable (approximately 18%) and the stimulation capability was not affected.

Despite the general resistance showed by ruminates to DON, the reproductive system seemed to be appreciably affected by this mycotoxin, based on in vitro studies. In this regard, the effects of DON on steroidogenesis have been investigated using, among others, the bovine granulosa cell model [73,109]. DON, in the presence of FSH and IGF1, was found to inhibit E2 production at concentrations ranging from 0.1 to 3.3 µM and P4 production at 0.33 and 3.3 µM [109]. In the absence of IGF1, DON at 3.3 µM significantly upregulated CYP19A1 mRNA abundance but had no effect on CYP11A1 mRNA abundance in bovine granulosa cells [73]. These results seem to support the theory that DON promotes stability of several mRNAs interfering with post-transcriptional processes and avoiding their rapid degradation with several adverse effects on steroidogenesis in cattle [73,109]. On the contrary, granulosa cell proliferation in the presence of FSH with or without IGF-I was not affected by DON (0.1–3.3 μM) [73].

#### 3.1.3. Poultry

Like bovine species, poultry seems to have greater tolerance to high doses of DON in terms of performance and productivity compared to other species. In poultry, a low level of absorption of DON into plasma and rapid metabolism and clearance from plasma was described. A study on oral bioavailability and toxicokinetics of DON reported that chickens do not hydrolyze DON3G to DON in vivo and that DON3G has a low absolute oral bioavailability (3.79 ± 2.68%) compared to DON (5.56 ± 2.05%) [110]. Moreover, intestinal microflora can convert DON to DOM-1 in poultry. Neither DON nor deepoxy-DON residues could be found in plasma and bile of broilers in an experiment by Danike et al. [111] in which DON concentrations in feed were approximately 1.5 mg/kg. The CONTAM Panel identified 5 mg DON/kg feed as a NOAEL for broiler chicken, since concentrations of 4.6–7 mg DON/kg feed did not cause adverse effects, while concentrations of 10–12 mg DON/kg feed caused signs such as reduced feed intake and reduced body weight gain. Based on the estimated feed concentrations, the risk of chronic adverse effects from the feed containing DON and its metabolites is low.

Acute DON mycotoxicosis in broiler chickens was characterized by extensive ecchymotic hemorrhages, deposition of urates, alteration of the nervous system, and inflammation of the upper gastrointestinal tract, but [112] this syndrome is unlikely to occur, due to the extremely high feed contamination involved. In another study, chickens were fed for 5 weeks with contaminated diet (1 mg and 5 mg DON/kg feed). The body weight, body weight gain, feed intake, and feed conversion were not altered by DON administration, but both concentrations tested significantly altered small intestinal morphology, particularly in the jejunum, where the villi were found significantly shorter [113].

Regarding egg production, no adverse effects were observed on egg production in terms of yield, egg weight and shell thickness [114,115,116,117], suggesting DON in this species does not affect ovarian follicular development. However, to our knowledge, no study has evaluated the direct effect of DON on avian granulosa cells.

In vivo and in vitro studies indicated that the immune system is sensitive to DON, which causes a reduction of lymphocytes, due to induction of the oxidative stress with DNA damage [118], and a reduction of the levels of vaccine antibody titers [119], confirming this system as a target for DON.

#### 3.1.4. Horses

In horses, DON could reach the systemic circulation only at low concentrations and undergo rapid clearance with glucuronide as the most represented form (approximately 80%) in plasma. For this species, the CONTAM Panel confirmed the previous NOAEL of 36 mg DON/kg feed for reduced feed intake [88]. Considering the dietary concentrations of DON and derived molecules, this mycotoxin is unlikely to represent a health concern for this species.

In a study by Johnson et al. (1997) [120], feed contaminated with 36–44 mg/kg DON was administrated to five healthy horses for 40 days. During this period, hematocrit values decreased in a linear progression, while peripheral white blood cells, polymorphonuclear leukocyte and lymphocyte counts as well as serum creatinine, sodium, potassium, chloride, total calcium, and inorganic phosphate concentrations were not changed. Serum activities of GGT, AST and creatine kinase decreased during the experimental period, and total serum protein, serum albumin and globulin, serum IgG and IgA did not show significant changes. None of the animals showed clinical signs of intoxication or reduced feed intake.

Regarding the effect on cellular and humoral immune parameters in horses, an experiment using naturally DON contaminated grains (high contamination −20.2 mg/kg and low contamination −0.49 mg/kg) was conducted by Khol-Parisini et al. [121] on two groups of five mares for two weeks. No adverse effects were observed; however, haptoglobin concentrations increased after feeding DON (*p* = 0.04). Lymphocyte counts and proliferation did not undergo changes indicating that DON does not impact the immune system in horses, confirming the high tolerance for DON in this species. To our knowledge, no studies have evaluated the direct effect of DON on equine granulosa cell function or reported any reproductive anomaly due to DON.

## 4. Zearalenone

Zearalenone (ZEA) is a phenolic resorcylic acid lactone mycotoxin produced by several *Fusarium* species, especially *F. graminearum*. Concern of ZEA occurrence in crops is related mainly to its contamination of maize kernels and maize products, although ZEA can contaminate other crops of agro-food importance. ZEA may undergo modification in plants, fungi and animals by phase I and phase II metabolism. Modified forms of ZEA found in feed include its reduced phase I metabolites (i.e., α-zearalenol, β-zearalenol, α-zearalanol, β-zearalanol, zearalanone) and its phase II conjugates (conjugate forms with glucose, sulfate and glucuronic acid). ZEA is rapidly and well absorbed after oral administration (uptake in a pig after a single oral dose of 10 mg/kg was estimated to be 80–85%) with an extensive biliary excretion. 

ZEA can undergo prehepatic, hepatic and extrahepatic metabolism and the predominant metabolic route and the amount of its metabolites are one of the reasons for different sensitivity to this toxin observed amonganimal species. While some differences have been reported on the hepatic biotransformation (pigs seem to convert ZEA predominantly into α-zearalenol, whereas in cattle β-zearalenol is the dominant metabolite) [9], a prevalence of reductive biotransformation was observed, leading to the production of compounds with retained or even increased metabolic activity.

It is already well established that ZEA and its metabolites have strong estrogenic activities, being able to cause alteration in the reproductive tract [13,122,123] acting as an endocrine disruptor. In particular, ZEA can induce estrogenic effects such as hyperestrogenism, anoestrus, ovarian atrophy and changes in the endometrium [122,124,125]. The effect of ZEA depends on several factors including the reproductive status (prepuberal, cycling or pregnant) of the affected animal and the administration time and dose [126,127,128,129]. A correlation between the level of ZEA in mg/kg and the length of anestrus in days was found by Young et al. [130], who observed an increase of the weaning-to-estrus interval when increased ZEA was fed.

ZEA acts by binding estrogen receptors (ERs), with a stronger affinity to ER-a compared to ER-b. There are differences between ZEA and its modified forms in terms of estrogenic action: a study performed in rats assessed the following rank: α-zearalenol > α-zearalanol > ZEA > zearalanone > β-zearalanol > β-zearalenol. As ZEA and its metabolites bind to oestrogen receptors as mixed agonists/antagonists, they are able to induce a syndrome described as hyperoestrogenism characterized by some typical signs such as edema of the vulva and mammary gland, vulvo-vaginitis, enlargement of the uterus, ovarian cysts, and impaired oocyte maturation [131]. Moreover, ZEA can activate the pregnane X receptor (PXR) and thus increase the transcription of many genes, including CYPs [132]. ZEA has also been reported to be hepatotoxic, haematotoxic, immunotoxic and genotoxic [133].

### 4.1. Species-Specific Effects

#### 4.1.1. Ruminants

In comparison to monogastrics, ruminants seem less susceptible to ZEA toxicity [73,134]. Although pigs are considered to be the most sensitive species, similar symptoms have been described in calves and young heifers [135], animals in which the ruminal system is not fully functional. When exposed to ZEA, cattle appear quite resistant and LOAEL or NOAEL could not be obtained.

The rumen protozoa convert ZEA into its hydroxy-metabolite forms α-zearalenol and β-zearalenol, even if ZEA also undergoes hepatic biotransformation [136]. Clinical signs due to ZEA ingestion (hyperestrogenism) are very rare in cows [106] and they occur only in cases of an extremely contaminated feed or after long-term exposure to contaminated feed materials [106] because of the poor rate of absorption of the ZEA metabolites. In cattle, another metabolite of ZEA, α-zearalanol, is used in some countries as a growth promoting agent [137].

A recent study of Pizzo et al. [73] determined the impact of α-zearalenol and β-zearalenol on granulosa cell function evaluating cell proliferation, steroid production and gene expression using a bovine granulosa cell model. Based on the results reported, in the absence of IGF1, α-zearalenol at 3.1 µM had inhibitory effects on cell proliferation, whereas it was found to inhibit both E2 and P4 production in granulosa cells at concentrations ranging from 0.09 to 3.1 µM in the presence of IGF1 [73]. Regarding β-zearalenol, an inhibitory effect on cell numbers was found at 31 µM both in the presence and absence of IGF1, while E2 and P4 production was increased in the absence of IGF1. The results obtained by Pizzo et al. on cell proliferation are in agreement with previous studies on pig granulosa cells conducted by Tiemann et al. [138] and Ranzenigo et al. [105], which demonstrated the adverse effects of these metabolites (see Section 4.1.2). In relation to the possible interaction between ZEA metabolites and gene expression, Pizzo et al. [73] demonstrated that α-zearalenol in the presence of IGF1 did not affect CYP11A1 and CYP19A1 mRNA abundance. Thus, α-zearalenol and β-zearalenol may impair bovine granulosa cell proliferation and steroidogenesis. In further support of an ovarian effect of ZEA, ewes fed ZEA (3–24 mg/ewe/day) during the estrous cycle had significantly reduced ovulation rates [139].

#### 4.1.2. Pigs

It is well known that ZEA exerts different harmful health effects in pigs, causing reproductive disorders, increasing oxidative stress, decreasing the nutrient digestibility while reducing growth rate [140]. Probably the high sensitivity to ZEA in this species is related to the fact that swine convert ZEA into α-zearalenol, which is a molecule more estrogenically active than its parent compound [125]. It is well established that prepubertal females are very sensitive to ZEA [141] and the guidance levels for ZEA in feed are 0.25–01 mg/kg depending on the category to which the pig belongs [142].

Symptoms are related to the dose of ZEA and the stage of estrous cycle in which the mycotoxin is consumed. The main targets of the oestrogenic syndrome in swine are the reproductive tract and mammary gland. In young gilts, 1–5 ppm of ZEA are sufficient to induce edema and hyperemia of the vulva, even vaginal and rectal prolapse [125], while, in cyclic animals, nymphomania, pseudopregnancy, ovarian atrophy and changes in the endometrium are more frequent [143]. Döll et al. [144] reported that after five weeks of feeding piglets in prepuberal status with feed contaminated by ZEA up to 0.42 mg/kg, the mean weight of the uteri was significantly increased. Recently, adverse effects on estrus were also reported [145]. This in vivo research provided evidence that ZEA at 1.04 mg/kg accelerated the development of the ovaries in post-weaning piglets, confirming that a diet contaminated by ZEA can accelerate the development of ovarian follicles in post-weaning piglets, possibly leading to subsequent reproductive disorders [145].

Because ZEA acts like estradiol (E2) and inhibits release and secretion of FSH, a suppression of follicle maturation occurs in the preovulatory stage.

The effects of α-zearalenol and β-zearalenol on pig oocytes have been investigated [146]. Oocyte maturation rate resulted in a significant decrease when oocytes were exposed for 48 h to α-zearalenol at concentrations up to 7.5 µM [146]. In contrast, β-zearalenol showed a significant effect only at 30 µM [146]. The ability of ZEA metabolites to affect steroid production in pig granulosa cells has also been reported by Ranzenigo et al. [105] and Cortinovis et al. [70]. In both studies, α-ZEA primarily increased progesterone (P4) production induced by FSH and IGF1, whereas E2 production exhibited a biphasic dose–response to α-zearalenol in the study conducted by Ranzenigo et al. and was not affected in the study of Cortinovis et al. Specifically, α-zearalenol at 0.094 µM and 9.4 µM increased and decreased E2 production, respectively [105], whereas no effects on E2 production were reported after exposure to α-zearalenol at 9.4 µM [70]. Regarding steroidogenesis, previous studies on pigs [105] showed an increase in E2 production at 0.09 µM, whereas, in cattle, no effects were observed at the same concentration [73], suggesting a species-specific effect. Moreover, previous studies in pigs [138] showed that α-zearalenol at 5 µM decreased CYP11A1 protein and gene expression. Similarly, Ranzenigo et al. [105] showed that α-zearalenol at 9.4 µM decreased CYP11A1 mRNA abundance in porcine granulosa cells. Thus, the predominant effect of α-zearalenol appears to be inhibitory to progesterone production in pigs, similar to the effects of estrogen on progesterone production by porcine granulosa cells [147].

#### 4.1.3. Horses

Toxicokinetics studies performed in vitro with primary cultures of equine hepatocytes and liver subfractions indicate that ZEA is mainly biotransformed into α-zearalenol, which was found at concentrations two- to three-fold higher than β-zearalenol. Then, ZEA and α-zearalenol underwent conjugation with glucuronide when present in low concentrations; otherwise, at higher concentrations, ZEA conjugation is prevalent [148]. In 1983, in Egypt, an outbreak of ZEA in horses was associated with corn containing approximately 2.6 mg/kg of this mycotoxin [149]. In a study performed by Aurich et al., six mares were fed with naturally contaminated oats (ZEA and DON at 1 and 12 ppm levels, respectively) and they found no significant effect on reproductive hormone release, cycle length and uterine histology [150].

Minervini et al. [151] investigated the effects of ZEA and its metabolites using an in vitro culture system of equine granulosa cells. The results of this study showed an increase in cell proliferation in the presence of ZEA (10^−3^ and 10^−4^ µM) and granulosa cell apoptosis after exposure to ZEA at 0.1 μM and α- and β-zearalenol. A recent study confirmed that ZEA induces necrosis and granulosa cell death in a dose-dependent manner via a caspase-3- and caspase-9-dependent mitochondrial pathway [152], indicating that these mycotoxins could be effective in inducing follicular atresia and altering reproductive function in mares.

## 5. T-2 and HT-2 Toxins

T-2 toxin (T2) and HT-2 toxin (HT2) are trichothecenes that belong to the type A group of trichothecenes. These mycotoxins are produced by various *Fusarium* species such as *Fusarium sporotrichioides*, and *Fusarium langsethiae* that infect crops in the field or during storage. HT-2 toxin is a natural contaminant in cereals but is also the main metabolite of T-2 toxin and the toxic effects of this mycotoxin are partially due to this metabolite. The toxicity of T-2 is influenced by many factors, such as the route of administration, dosage and moment of exposure, age, sex, general physical condition of the exposed animal and of course co-exposure [153].

As the other trichothecenes, T-2 toxin inhibits protein, RNA and DNA synthesis, induces apoptosis and necrosis in some cell types and lipid peroxidation, which threaten the cell membrane integrity. Regarding protein synthesis, in vitro studies suggest that T-2 toxin interacts with the peptidyl transferase of the 60S ribosomal subunit inhibiting the formation of the new peptide-bond formation [154]. In addition, T-2 toxin induced apoptosis in various cell lines (i.e., Vero cells and human hepatoma cells [155,156]. Similarly, T-2 toxin induced apoptosis was also reported in in vivo studies involving haematopoeitic-lymphoid tissues, brain and skin [157,158]. One of the mechanisms involved in the induction of apoptosis caused by this mycotoxin could involve the activation of caspases as observed in human promyelocytic leukemia cells [159]. However, the mechanisms by which T-2 toxin induces apoptosis is still not fully understood; the more reliable hypothesis indicates that the DNA damage, which could be a secondary effect of protein synthesis inhibition or a direct effect mediated by the oxidative stress, can activate mitochondrial apoptotic pathways [154]. In addition, the apoptosis could be linked to the activation, caused by the protein inhibition or through ROS production, of stress-activated protein kinase (SAPK/JNK) and mitogen activated protein kinase (p38/MAPK) [10]. T-2 toxin is able to cause lipid peroxidation with the production of ROS because of its amphophilic nature that allows the molecule to be incorporated into the bilayer membranes. Then, radicals are generated causing lipid peroxidation with cellular membrane damages and oxidative stress of the cells [160].

The immune system is one of the main targets of T-2 toxin showing an immunomodulatory activity, e.g., it can stimulate (low dosages of the toxin) or inhibit (high dosages) activity of the immune system in a time- and dose- dependent manner. Apoptotic effects in Peyer patches, mesenteric lymph nodes and thymus have been described in mice by Nagata et al. [161] 24 h after a single oral administration (10 mg/kg). Moreover, T-2 toxin perturbs the maturation of the antigen-presenting cells by altering lymphocyte proliferation antibody levels leading to an increased susceptibility to infectious diseases.

Summarizing, the main effects of trichothecene mycotoxins on exposed animal species are reduced feed intake and weight gain, gastrointestinal disturbances, neuroendocrine and hepatological changes and impairment of the immune system. Moreover, T-2 toxin showed a local irritant effect and caused serious hemorrhagic inflammation due to a damaging effect exerted on the blood vessel walls with hemorrhagic diathesis. Other lesions described in literature are necrosis and ulceration in the digestive tract, alteration in kidney, heart, brain and peripheral ganglia of the vegetative nervous system [162].

### 5.1. Species-Specific Effects

#### 5.1.1. Ruminants

Ruminants are considered less sensitive to the effects of T-2 toxin because of the role played by rumen in detoxification. In this regard, young animals, with a rumen not fully functional, could show higher susceptibility to this toxin and exposures to 0.3 mg T-2 toxin/kg body weigh per day could result in gastrointestinal lesions, altered serum proteins and hematological alterations in calves.

Intoxication in adults are rare, but, in one study in 1972, seven cows died with massive hemorrhages of the gastro intestinal tracts and this episode was linked to T-2 intoxication [163]. Consumption of feed contaminated at a level of 640 ppb (ppm = 1000 ppb) for a period of three weeks could cause bloody feces and abomasal and ruminal ulcers, which may lead to death, but such a large contamination is infrequent. In terms of reproductive effects, T-2 toxin was fed to ewes at 0.9 mg/day and heifers at 9 mg/day and found that T-2 toxin caused reduced plasma progesterone and delayed ovulation [164] implying that this toxin may reduce reproductive performance in ruminants. To our knowledge, no study has evaluated direct effects of T-2 toxin on ovine or bovine granulosa cells in vitro.

#### 5.1.2. Pigs

All trichothecenes are known to reduce reproductive performance in pigs [165,166]. However, pigs are among the most susceptible animals towards the effects of T-2 toxin. Immunological and/or hematological effects occur starting from doses of 29 μg T-2 toxin/kg b.w. per day. Prolonged exposure to type-A trichothecenes, and particularly T-2 toxin, was associated with anorexia, reduced body weight gain and lesions of oral cavity and esophagus. Moreover, T-2 toxin decreases protein synthesis, impairs the immune response inducing leucopenia, induces cell depletion in lymphoid organs impairing antibody production, and inhibits erythropoiesis [167].

In a study performed by Caloni et al. [166], porcine granulosa cells were cultured to evaluate the influence of T2 on steroid production and cell proliferation. The research showed that T-2 toxin had potent inhibitory effects on IGF-I and FSH-induced steroid production: dosages of 1, 3, 30 and 300 ng/mL completely inhibited estradiol production, which appeared to be more sensitive to the inhibitory effect of T-2 toxin than progesterone was (progesterone production was completely inhibited with a dose of 30 and 300 ng/mL). T-2 toxin also had an inhibitory effect on cell number at 3 ng/mL. These findings indicate that T-2 toxin may be able to alter the growth of the granulosa cell layer as well as the steroidogenesis with potent direct dose-dependent effects.

In another study performed by Zhang et al. [168], the toxic effects of HT-2 on porcine oocyte maturation were investigated in vitro, treating porcine oocyte with HT-2 toxin. The addition of the toxin inhibited the cumulus cell expansion and the polar body extrusion with disruption of meiotic spindle probably because of a reduction in the p-MAPK protein level. Actin distribution also was perturbed, indicating that HT-2 can alter the cytoskeleton of porcine oocytes. Moreover, treated oocytes showed an increased ROS level, a marker of oxidative stress. 

Collectively, these studies confirm the potential of T-2 toxin and its metabolites as disrupters of reproductive performance of pigs.

#### 5.1.3. Poultry

In poultry, the T-2 toxin has genotoxic and cytotoxic effects, immunomodulatory effects, effects on the cells of the digestive system and liver, effects on the nervous system and skin and impairment of performance. The T-2 toxin is quickly absorbed in the intestinal tract of chickens, then metabolized, and almost completely (about 90%) eliminated in one day even if entero-hepatic recirculation has been described and could increase and prolong the toxic effects. Detoxification of T-2 toxin and related trichothecenes due to intestinal microflora has not been demonstrated in this species [169].

The first signs of T-2 toxicosis are lower feed intake, reduced weight gain and growth retardation or lower egg production (reported, for dosages of 1–10 mg/kg, of 12.5–78.8% respectively) [170], lower egg weight, thinner egg shells (only with high dosages of 20 mg/kg) [171] and decreased hatchability [169]. The reduction in egg production in this and other studies [170,172,173] suggests that T-2 toxin may impact ovarian follicular growth, but direct effects of T-2 toxin on granulosa cell function has not been reported in chickens. The lethal dose of T-2 toxin in feed during a feeding period of seven days was about 10 mg/kg of chicken body weight [174].

Other reported symptoms are neurologic signs, leucopenia, mouth lesions (sometimes a single dose of 5 mg/kg is sufficient to cause this lesion, but more frequently long-term administration of contaminated feed with 1 to 5 mg/kg of toxin is needed), cyanosis of the comb, depigmentation of the leg skin and changed feather quality. The results of histopathological examination often reported the detection of the characteristic necrotic lesions (characterized by white-yellowish aspect of the mucosa also due to the presence of caseous-necrotic material) in mouth, crop, gizzard, intestinal mucosa and the liver. These target organ effects and immune system effects are a result of protein synthesis inhibition that causes a reduced activity of the enzymes responsible for toxicant metabolism and induction of lipid peroxidation [175].

#### 5.1.4. Horses

Limited published information is available on the effects of T-2 toxin on this species. In 1986, 30 horses fed with contaminated cereals (containing 204 mg T-2 toxin/kg) showed signs of mycotoxin intoxication. Twelve animals started to show locomotive alteration and then died after a month. Blood chemistry showed severe alteration (leukocytosis, anaemia) and liver underwent fatty degeneration [176]. Later, the effects of long-term administration were studied in six trotter mares given a daily oral dose of 7 mg of purified T-2 for 32–40 days. During the experiment, horses remained in good general condition but some skin lesions were observed around the mouth in three animals. No effects on the length of the interovulatory interval, the luteal and follicular phases and fertilization were detected in these mares. T-2 administration also had no effect on peripheral plasma progesterone profiles and follicular kinetics. Moreover, embryo recovery by uterine flush was performed in five mares obtaining three embryos with a percentage of success of 60%, which is the normal expected percentage. The embryos exhibited normal size and morphology [177].

These results indicate that administration T-2 toxin to mares, at the dose tested, has no detrimental effects on reproduction. To our knowledge, no study has evaluated the possible direct effect of T-2 toxin on equine granulosa cell function.

According to Raymond et al. [178], potentially significant levels of DON and T-2 toxin can be found in 50% of samples of field-dried hay fed to performance horses. Later, Liesener et al. [179] demonstrated the co-occurrence of DON and T-2 toxin in commercial horse feed, indicating the real risk of exposure to these toxins in horses. In this regard, a possible correlation between the presence of DON and T-2 and the incidence of colic in horses has also been described [180] and, in competing horses, the exposure even if at low levels may be detrimental for performance and breeding activities, without clinical signs of toxicity [181].

## 6. Beauvericin

Beauvericin (BEA), produced by a wide range of *Fusarium* species, is considered for its toxicity as a minor *Fusarium* mycotoxin, if compared to the above-mentioned mycotoxins. However, BEA represents a major concern because of its potential toxicity in humans and animal health, and its high presence in feed and food commodities in co-occurrence with several other *Fusarium* mycotoxins.

BEAs, as well as enniatins, are cyclic depsipeptides. This structure is characterized by the presence of free electron pairs of the oxygen-carbonyl groups and tertiary amino groups that can form ion–dipole interactions, acting as nucleophiles [182]. Moreover, the absence of stabilizing intramolecular hydrogen bonds due to the N-methylation of amino acid parts gives flexibility to the molecule. Because of this, BEA is unstable and can form complexes with many metallic cations (i.e., sodium, potassium, calcium, barium, etc.) and many other molecules, even neutral ones [183]. The toxic effects of BEA are above all a consequence of its molecular structure, which gives to the toxin its ionophoric properties leading to the capacity to act as an ion carrier throughout the cytoplasmic membrane [184]. Other mechanisms of action involve enzyme inhibition. For example, Tomoda et al. demonstrated that BEA is able to inhibit acyl-CoA cholesterol acyltransferase (ACAT), an enzyme that allows the production of the cholesteryl esters from cellular cholesterol and long-chain fatty acyl-CoA [185], and others have reported the capacity to induce oxidative stress with the production of ROS [186]. For example, Caco-2 cells treated with 1.5 or 3 μM BEA produced ROS in a dose- and time-dependent manner [187]. Moreover, the generation of ROS is followed by a higher lipid peroxidation and a decrease in the glutathione level [186], probably as an antioxidant system. As a result of all these actions, BEA can perturb the physiological intra- and extracellular ion concentrations and induce apoptosis in many cellular lines.

The mechanisms by which BEA can induce apoptosis have been investigated. Apoptosis is likely due to the ability of BEA to increase the intracellular concentration of calcium, which leads to the activation of Ca-dependent endonuclease [188]. Other pathways involved in cellular apoptosis may include a loss of membrane potential of the mitochondria with release of cytochrome and activation of the enzyme caspase 3, which plays a central role in the execution phase of the cell apoptosis [189].

BEA, in addition to its cytotoxic properties, displays anticancer [190,191], antimicrobial [192], insecticidal [193] and nematocidal actions [194]. The latter action is probably related to the non-competitive inhibition of the calcium channels of parasites’ smooth muscle, while, regarding the anticancer effects, they have been identified in several cancer cell lines. In a study by Heilos and collegaues [190], the specificity of the anticancer activity was verified in vitro comparing the effects exerted by BEA on malignant and non-malignant cells. The anticancer effects of BEA were also assessed in vivo, treating mice bearing murine CT-26 or human KB-3-1-grafted tumors. The results showed a more intense cytotoxic activity against malignant cells and a decreased tumor volume and weight in BEA-treated mice compared to controls, and this was found both in allograft and in xenograft models. Moreover, the necrotic areas within the tumor, as well as the apoptotic cells, were increased in mice administered with BEA compared to controls. Regarding BEA’s effect as an endocrine disruptor, a reduction in the transcriptional activity of the androgen, glucocorticoid, oestrogen and progestagen receptors was observed at a concentration of 10 µM. An antagonistic effect of BEA was observed on the progestagen and glucocorticoid receptors at non-toxic concentrations (1 µM). Moreover, BEA significantly decreased cell viability at 10 µM, and, at the same dosage, induced significant toxicity in both the TM-Luc progestagen responsive cells and Caco-2 cells [71].

Very few data regarding the BEA species-specific toxicity are available and most of the studies were conducted in vitro. Most of the in vivo experiments were performed on birds via administering naturally contaminated feedstuff [195,196], so the specific action of BEA is difficult to identify.

### Species-Specific Effects

An in vitro study, performed by Albonico et al. [69], used bovine granulosa cells to evaluate the effects of BEA on cell proliferation, steroid production and gene expression. The BEA-induced inhibition of estradiol production after 48 h of treatment (dose equal or >3 µM) was associated with a significant decrease in CYP19A1 mRNA abundance after 24 h (at a dose of 30 M), thus it is likely that BEA may alter estradiol production via suppression of CYP19A1 mRNA abundance. In the same way, the BEA-induced inhibition of progesterone production after 48 h of treatment was associated with a significant suppression of CYP11A1 mRNA abundance after 24 h, so BEA may perturb progesterone production via acting on CYP11A1 mRNA abundance.

BEA also decreased bovine granulosa cell proliferation [69] at concentrations > or equal to 6 µM, confirming its cytotoxicity, as reported in previous studies using other mammalian cell lines [197,198].

An in vitro study by Santos et al. [199] exposed porcine granulosa cells to BEA and found that a concentration of 5 μM led to the upregulation of ABCG2 gene expression (the membrane-associated protein encoded by this gene is included in the superfamily of ATP-binding cassette (ABC) transporters that transport various molecules across extra- and intra-cellular membranes) and to the downregulation of CYP19 expression. Moreover, Santos and colleagues confirmed the BEA cytotoxicity observed in bovine granulosa cells. Collectively, the few studies that have evaluated direct effect of BEA on granulosa cell function suggest that BEA may inhibit ovarian function and thus be a detriment to reproductive performance in pigs and cattle. More studies are needed to determine potential species differences in BEA effects.

## 7. Enniatin B

Enniatins (ENNs) are mycotoxins produced by several *Fusarium* species. Structurally, ENNs are cyclohexadepsipeptides, and many ENN analogs have been identified [200], but, among them, the most important in terms of incidence and the most studied is the ENN B. Despite the emerging interest in this toxin, as well as in BEA, their toxic profiles are still almost unknown. An in vivo study performed in mice [201] showed that, after intraperitoneal administration of ENN B and BEA, these toxins can be identified in all tissues evaluated and in serum. The highest concentrations were found in adipose tissue and liver, revealing a tendency for these compounds to bioaccumulate in lipophilic tissues. Regarding the metabolism of ENN B, while several phase I metabolites (namely dioxygenated-ENN B, mono- and di-demethylated-ENN B) were found in liver and intestine, no mycotoxin or metabolite presence was found in urine, thus it could be hypothesized that hepatic and intestinal metabolism could be preeminent. Conversely, no metabolites were identified for BEA.

The enniatin molecules, as with BEA molecules, do not have any ionic group but have free electron pairs that can act as nucleophiles leading to weak chemical interactions with cations. Because of this particular three-dimensional structure, ENN can act as ionophores showing both hydrophilic and hydrophobic properties. Thus, ENN could be incorporated into the lipid bilayers of cell membranes acting as selective pores and increasing the permeability for alkali cations. This may lead to the alteration, as for BEA, of the intra and extra-cellular environment [202,203].

As for BEA, the mechanisms of action of ENN B include the induction of oxidative stress, the inhibition of acyl-CoA cholesterol acyl transferase (ACAT) enzyme, action on ABC transporters and apoptosis induction via alteration of the mitochondrial functions. The mitochondriotoxic action of this toxin is probably due to the cation ionophoric activity that allows K^+^ influx into the mitochondria [202] and the Ca^2+^ efflux, the latter through the permeability transition pore (PTP) that traverses both the inner and outer mitochondrial membrane causing the loss of the mitochondrial membrane potential [203].

Primarily, a lot of the biological activities of this molecule are directly related to its ionophoric behavior. ENN showed cytotoxic, antibacterial, antifungal, insecticidal and antihelmintic properties [52]. Recently, ENNs have been demonstrated to exert a potential anticancer activity being able to avoid the expulsion by ABC transporters and targeting some tumor cell types [204]. More studies are needed to better understand the species-specific action of these mycotoxins. To date, human colon intestinal Caco-2 cells have been the most used cell line in studies that applied ENN B alone, but ENN also proved to exert cytotoxic effects against several mammalian cell types [203,205], at quite low dosages (micromolar concentration).

Some of these in vitro studies evaluated ENN B alone and mixtures of ENN because co-exposure, due to the contemporary production of different mycotoxins even from the same mold species, is likely to occur.

Compared to in vitro studies, there are few in vivo studies. Considering the high occurrence of these mycotoxins in feed products, possible risk through the ingestion and the in vivo and in vitro species-specificity of the effects of ENN need to be more thoroughly investigated. A recent study performed by Rodríguez-Carrasco and colleagues [206] evaluated, for the first time, the occurrence of ENN B and its metabolites in human urine samples obtained from an Italian population (Campania region). Among the 300 examined samples, 83.7% of them were positive for ENN B presence. Similarly, ENN B metabolites were found in 6.7% to 96.3% of the samples, depending on the type of metabolite investigated. These data indicate that ENNB exposure in southern Italy is frequent and likely to occur, and that more studies are needed to better understand the implication of this widespread emerging mycotoxin.

### Species-Specific Effects

No reports are available regarding acute ENN mycotoxicoses in human and animal species [52]. Therefore, possible effects due to chronic exposure of ENN at subclinical levels may be of greater importance since it may cause reduced performance parameters in food producing animals and increased susceptibility to infectious diseases. The limited in vivo toxicity might result from the toxicokinetic characteristics of this compound, but this needs to be investigated deeply, even though it seems related to the rapid elimination of the mycotoxins. The absolute bioavailability of ENN B1 in pigs was 91% after single oral administration of 0.05 mg/kg body weight, with higher plasma concentrations reached after 15 min, indicating a rapid gastrointestinal absorption [207].

Regarding the intravenous route of administration, it was characterized by a high clearance and moderate volume of distribution, following the same pattern observed in chicken [207,208]. The main metabolites of ENNs are phase I metabolites, as in vitro and in vivo studies have clarified, even if profiles may differ between species following species-specific pathways. Considering this, detailed studies on species-specific metabolite formation and in vitro and in vivo correlation would be useful.

To assess exposure and in vivo toxicity, the toxicokinetic properties were investigated in several works. A study by Ivanova et al. [209] described the metabolism of ENN B1 in swine both in vitro and in vivo, comparing in vitro metabolites obtained using liver microsomes from different pig strains to those found in plasma after a single oral or intravenous ENN B administration. The metabolite formation was higher when ENN B1 was administrated orally than intravenously indicating a pre-systemic metabolism of the toxin after oral uptake. In vitro culture allowed the detection of 10 ENN B1 metabolites, while just six were detected in vivo. Metabolites were hydroxylation, carbonylation, carboxylation and oxidative demethylation products.

Studies on ENN kinetics were also performed in chickens. Fraeyman et al. [208] performed an experiment on broilers chicken administering orally and intravenously 0.2 mg/kg body weight of ENN B or B1 and found that ENN B1 as well as B were poorly absorbed after oral administration, with absolute oral bioavailabilities of 0.05 and 0.11, respectively. Both enniatins were readily distributed to the tissues, with mean volumes of distribution of 25.09 and 33.91 L/kg. The mean total body clearance was high (6.63 and 7.10 L/h/kg). Regarding the metabolites, either glucuronide nor sulfate phase II metabolites were detected, with oxygenation as a main phase I biotransformation pathway for these ENNs. This study showed different results compared with those obtained in swine species, confirming the species-specificity of the metabolic pattern of this mycotoxin and suggesting the need of specific investigations that evaluate the particular metabolites of the different species.

## 8. Conclusions

The broad range of toxic activities that the *Fusarium* mycotoxins, worldwide contaminants of food and feed, exerted on animals and humans are a source of a great concern, potentially amplified by the co-occurrence of more than one mycotoxin in the same commodities. Examples of this risk are represented by *F. graminearum* that can produce DON and ZEA together, and *F. proliferatum* that produces FB1, BEA, ENNs and other mycotoxins. On the other hand, most of the time, the colonization of crops by *Fusarium* species is a result of complex attacks where more than a single species is involved. FHB of wheat and FER of maize are well-known diseases caused by the colonization of multiple *Fusarium* species. The need to evaluate more carefully the toxicity expressed by the combination of two or more *Fusarium* mycotoxins (and their species-specific metabolites) is a challenge for future toxicological studies with particular interest considering possible health effects on animals, and on humans.
